# Supplementary figures and images for: The Signal and the Noise: Characteristics of Antisense RNA in Complex Microbial Communities
Source: mSystems. 2020 Feb 11;5(1):e00587-19. doi: 10.1128/mSystems.00587-19 (PMC7018526; doi:10.1128/mSystems.00587-19)

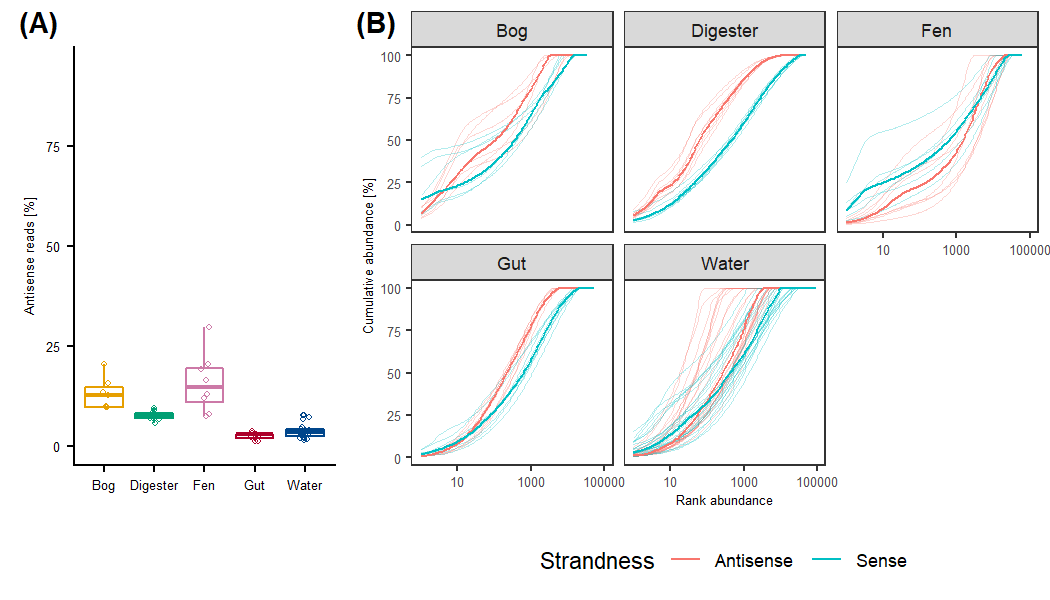

Supplement: FIG S1 [file mSystems.00587-19-sf001.tif]

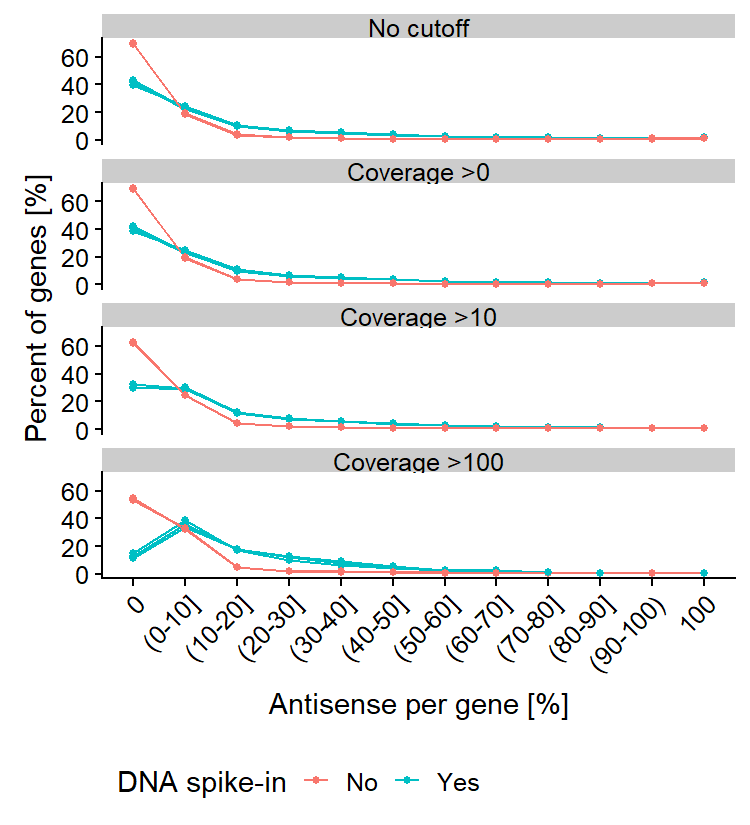

Supplement: FIG S3 [file mSystems.00587-19-sf003.tif]

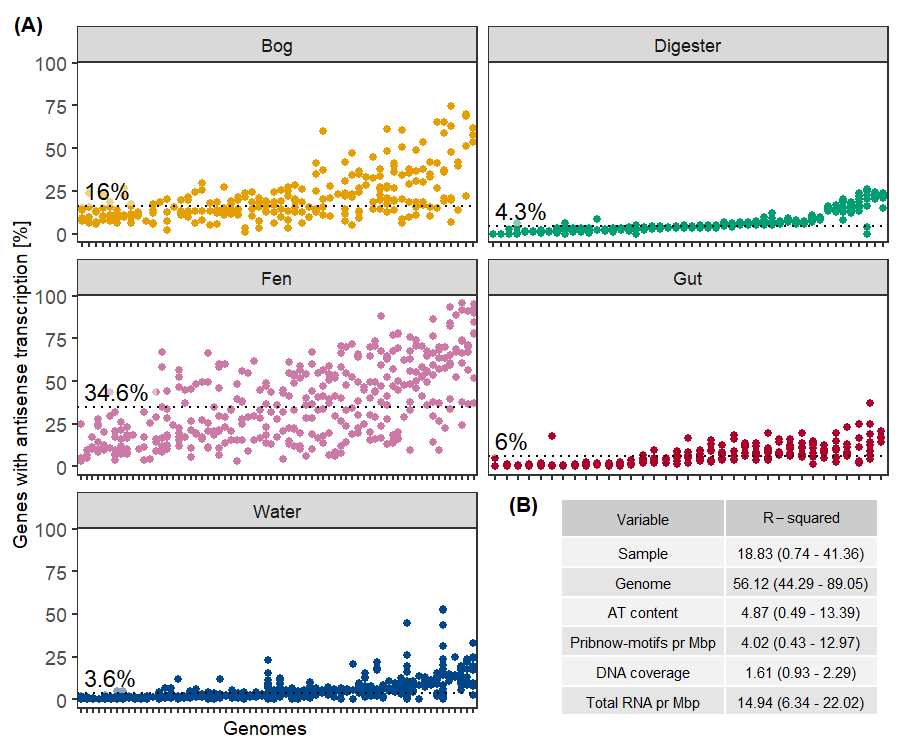

Supplement: FIG S2 [file mSystems.00587-19-sf002.tif]
