# Supplementary material for: The Signal and the Noise: Characteristics of Antisense RNA in Complex Microbial Communities
Source: mSystems. 2020 Feb 11;5(1):e00587-19. doi: 10.1128/mSystems.00587-19 (PMC7018526; doi:10.1128/mSystems.00587-19)
Supplement: TABLE S1 [file mSystems.00587-19-st001.docx]

|  | **Digester** | **Bog** | **Human gut** | **Water** | **Fen** |
| --- | --- | --- | --- | --- | --- |
| **Samples** | 9 | 7 | 8 | 24 | 8 |
| **Genomes/MAGs** | 51 | 69 | 62 | 74 | 92 |
| **AT content (%)** | 53 (40 - 71) | 40 (29 - 68) | 57 (32 - 72) | 49 (28 - 75) | 52 (32 – 68) |
| **Size (Mb)** | 2.3 (1 - 3.9) | 4.3 (1.5 – 8) | 3.3 (1.8 - 6.3) | 2.9 (0.7 - 7.7) | 3.2 (1.5 – 8.7) |
| **Genes** | 2089 (1006 - 3394) | 3868 (1622 – 7176) | 2830 (1591 - 4902) | 2729 (768 - 6573) | 3039 (1622 – 8069) |
| **Completeness (%)** | 88 (57 - 98) | 95 (71 – 100) | 99 (76 - 100) | 89 (51 - 99) | 90 (71 – 100) |
| **Contamination (%)** | 1 (0 - 10) | 2 (0 – 9) | 0 (0 - 3) | 1 (0 - 10) | 2 (0 – 9) |

Table S1 | Characteristics of MAGs from the five different environments.
